# Cerebral Small Vessel Disease: Bystander or Culprit?

**DOI:** 10.7759/cureus.96026

**Published:** 2025-11-03

**Authors:** Emma L King, Hla Hla Aye, Eluzai Abe Hakim

**Affiliations:** 1 Emergency Department, University Hospitals Dorset NHS Foundation Trust, Bournemouth, GBR; 2 Cardiology Department, University Hospitals Dorset NHS Foundation Trust, Bournemouth, GBR; 3 Stroke Medicine Department, University Hospitals Dorset NHS Foundation Trust, Bournemouth, GBR

**Keywords:** cadasil, cerebral small vessel disease, dementia, migraine, notch3, stroke

## Abstract

Cerebral small vessel disease (SVD) contributes to about 45% of dementias and causes substantial cognitive, psychiatric, and physical disability. The most common form of monogenic strokes is cerebral autosomal dominant arteriopathy with subcortical infarcts and leukoencephalopathy (CADASIL), which is caused by mutations in the NOTCH3 gene. This leads to cerebrovascular NOTCH3 protein aggregation and compromises cerebral blood flow. We present the case of a 69-year-old female patient presenting with dysarthria, a past medical history of migraines, and a family history of early-onset stroke. She was treated at a large district general hospital. Non-contrast computed tomography (CT) brain showed changes in keeping with small vessel ischaemic change. Subsequent magnetic resonance imaging (MRI) revealed an acute right frontal lobe infarct with extensive high signal intensity changes. Involvement of the temporal lobes suggested CADASIL as a possible differential diagnosis. The genetic testing confirmed a heterozygous pathogenic NOTCH3 variant. This case highlights the importance of testing for CADASIL in patients with extensive SVD changes coupled with a history of stroke at an early age or family history of strokes. Informed written consent was obtained from the patient to publish her clinical information anonymously.

## Introduction

Cerebral small vessel disease (SVD) of the brain is a term used to describe a syndrome of clinical, cognitive, neuroimaging, and neuropathological findings thought to arise from the disease affecting the perforating arterioles, capillaries, venules, and the resulting brain damage in the cerebral white matter of the brain [1]. The disease is very common and causes substantial cognitive [2], psychiatric [3], and physical disabilities [4]. It contributes to about 45% of dementias [5].

SVD is not a single disease but can be caused by diverse pathological processes, the most common of which are hypertension, arteriosclerosis caused by ageing, conventional risk factors, and cerebral amyloid angiopathy due to vascular deposition of beta amyloid. Rarer causes include monogenic conditions such as cerebral autosomal dominant arteriopathy with subcortical infarcts and leukoencephalopathy (CADASIL) [6]. CADASIL is caused by mutations in the NOTCH3 gene. NOTCH3 gene mutations are diagnostic for the most common hereditary cerebral small vessel disease, which is estimated to occur at a frequency of 2 to 5 per 100,000 persons in the general population worldwide [7]. NOTCH3 mutations in CADASIL lead to (cerebro)vascular NOTCH3 protein aggregation and compromised cerebral blood flow, with more than 200 distinct NOTCH3 mutations identified in CADASIL [8,9]. The majority of individuals with a NOTCH3 variant will develop NOTCH3-associated SVD after the age of 65; therefore, NOTCH3 should be considered as a genetic risk factor in SVD risk stratification and prevention [9].

Clinical presentations of patients with CADASIL are variable, with a constellation of symptoms including migraine, psychiatric disorders, cerebral vascular events, and progressive dementia [10]. We report a case of a 69-year-old female patient who was admitted to the stroke unit of a district general hospital with slurred speech, episodes of migraine, and symptoms suggestive of transient ischaemic attack or stroke.

## Case presentation

A 69-year-old female patient, with a past medical history of hypertension and migraine with aura, presented to the emergency department of a general district hospital with slurred speech. The dysarthria had developed three days prior to presentation and had not worsened. She also noticed drooling of saliva from the left angle of her mouth. She was not on any regular medications. Her mother suffered a stroke at 40 years of age. She lived with her husband and was fully independent. She did not use tobacco products and occasionally drank alcohol. At admission, full blood count (FBC), urea and electrolytes (U&Es), C-reactive protein (CRP), bone profile, and liver function tests (LFTs) showed no abnormalities.

**Figure 1:**
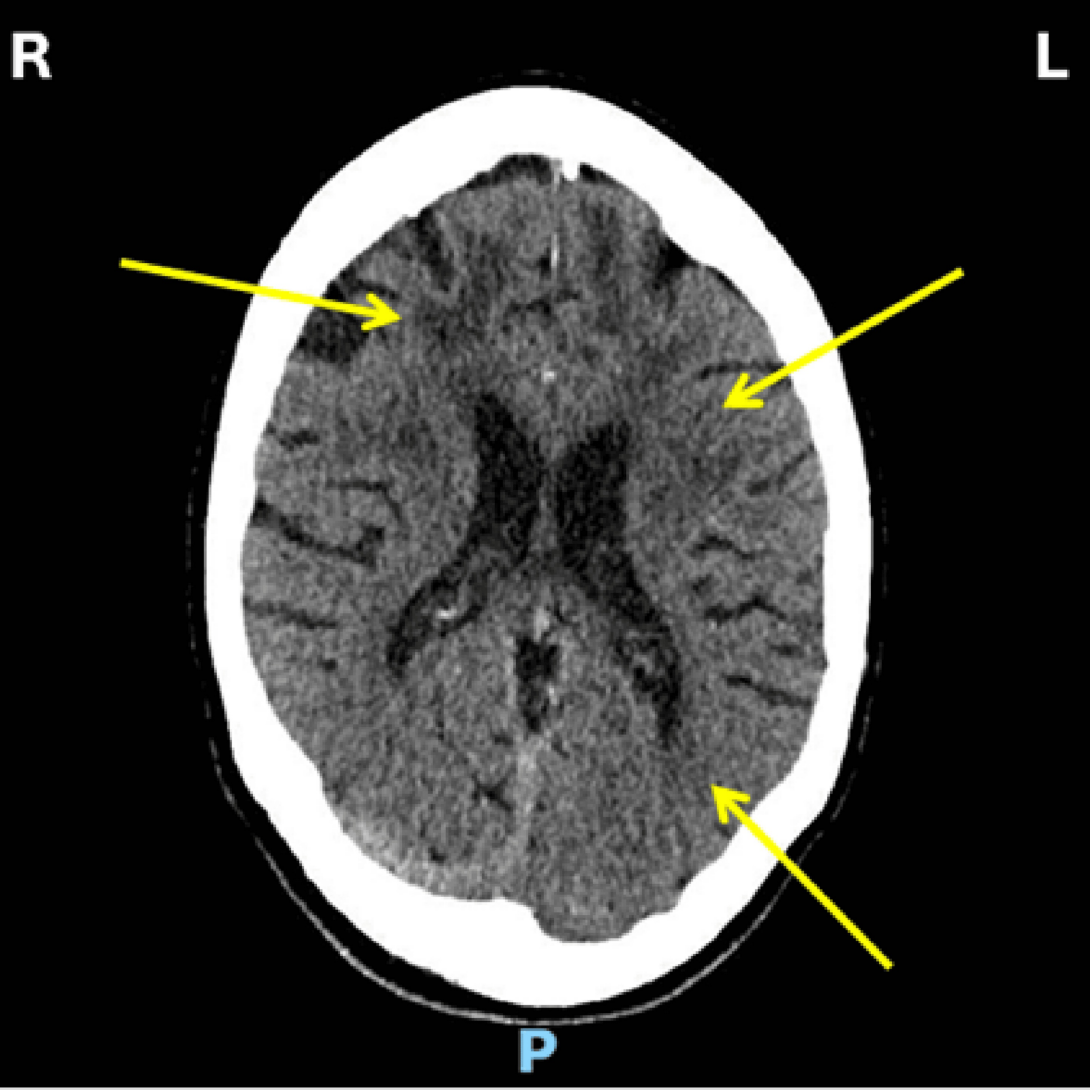
Axial non-contrast CT head demonstrating small vessel disease (yellow arrows) CT: computed tomography CT head showing confluent white matter low attenuation periventricular areas in keeping with small vessel disease ischaemic change

Clinical examination elicited a left-sided facial droop of upper motor neuron distribution and mild dysarthria. No other abnormal focal neurological findings were elicited on the examination of the neurological system. The cardiovascular, respiratory, and abdominal systems were unremarkable. The National Institutes of Health Stroke Scale (NIHSS) on admission was one for mild dysarthria. A clinical diagnosis of right hemispheric lacunar infarct was made. Non-contrast brain computerised axial tomographic scan (CT) showed no acute intracranial abnormality but confluent white matter low attenuation in the periventricular areas in keeping with small vessel disease (Figure 1).

She was given a loading dose of 300 mg of aspirin, and dual antiplatelet therapy with 75 mg of aspirin and 75 mg of clopidogrel was commenced. Aspirin was scheduled for 21 days as per hospital protocol, and lansoprazole 30 mg, orally, daily, was prescribed for gastroprotection during the duration of the aspirin intake. Clopidogrel, 75 mg, orally, daily, was continued lifelong for secondary protection.

**Figure 2:**
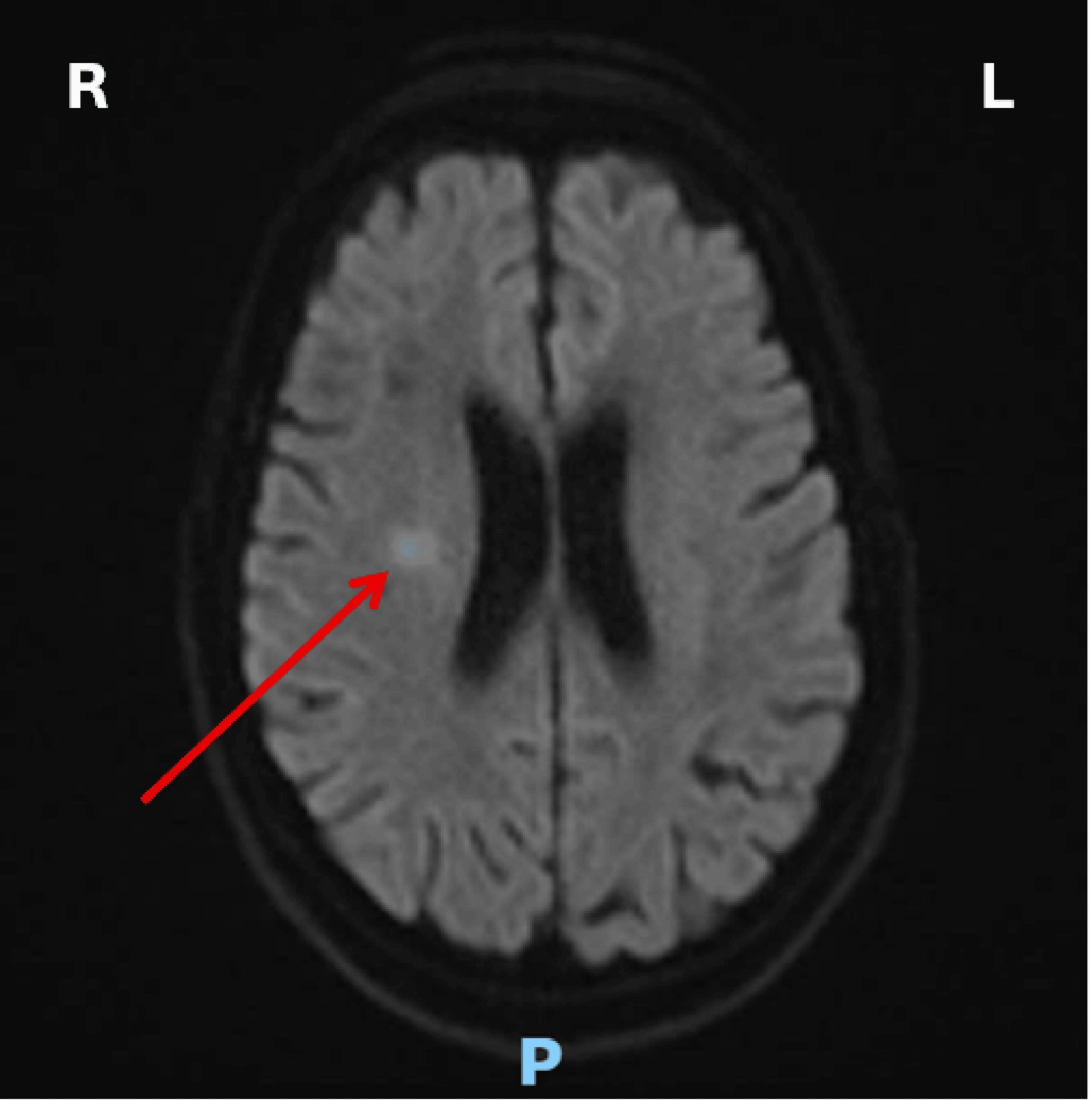
Axial MRI brain DWI demonstrating an acute infarct (red arrow) MRI: magnetic resonance imaging; DWI: diffusion-weighted imaging MRI brain showing a small acute infarct in the white matter of the right frontal lobe

**Figure 3:**
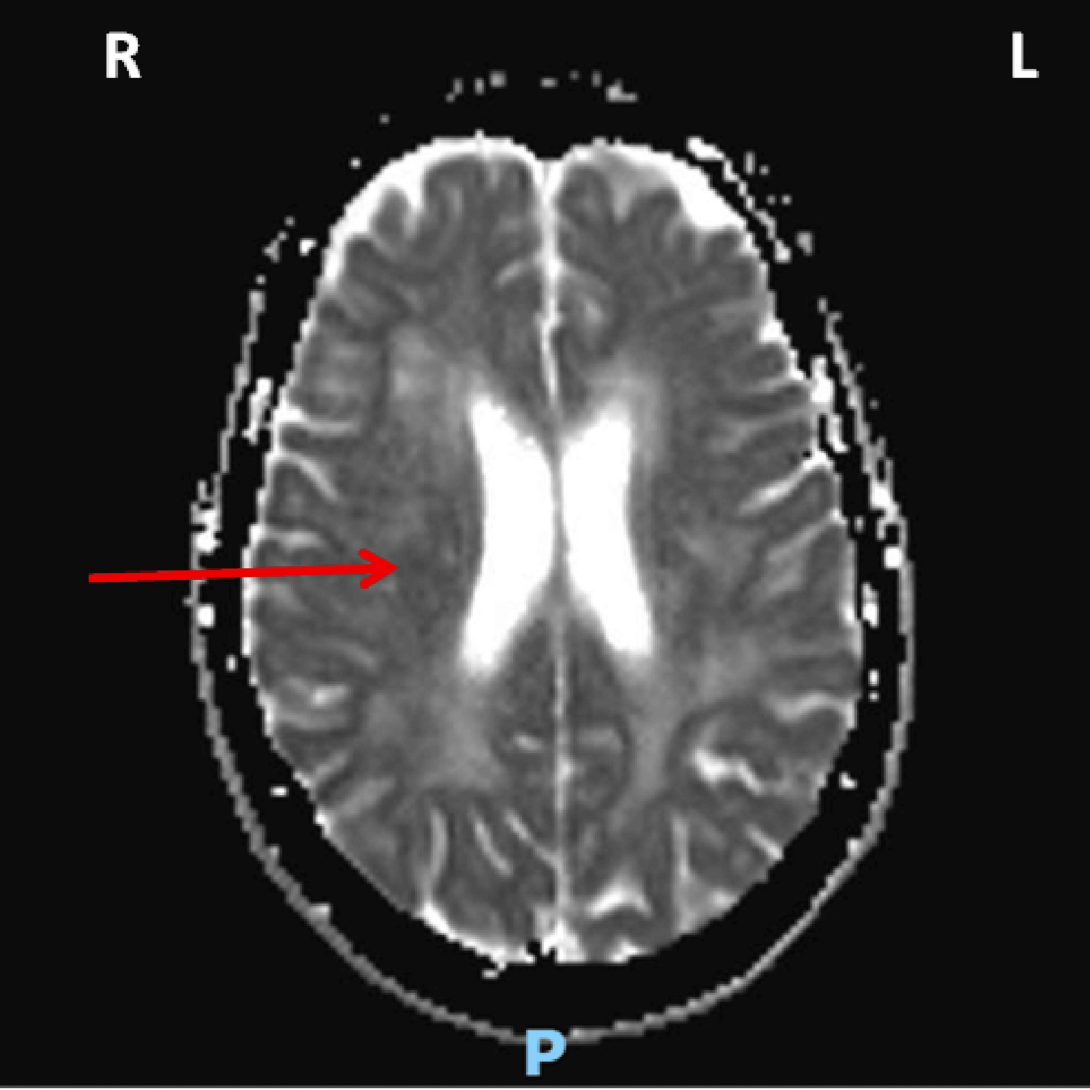
Corresponding lesion shown on axial MRI brain ADC confirming an acute right frontal lobe infarct (red arrow) MRI: magnetic resonance imaging; ADC: apparent diffusion coefficient

**Figure 4:**
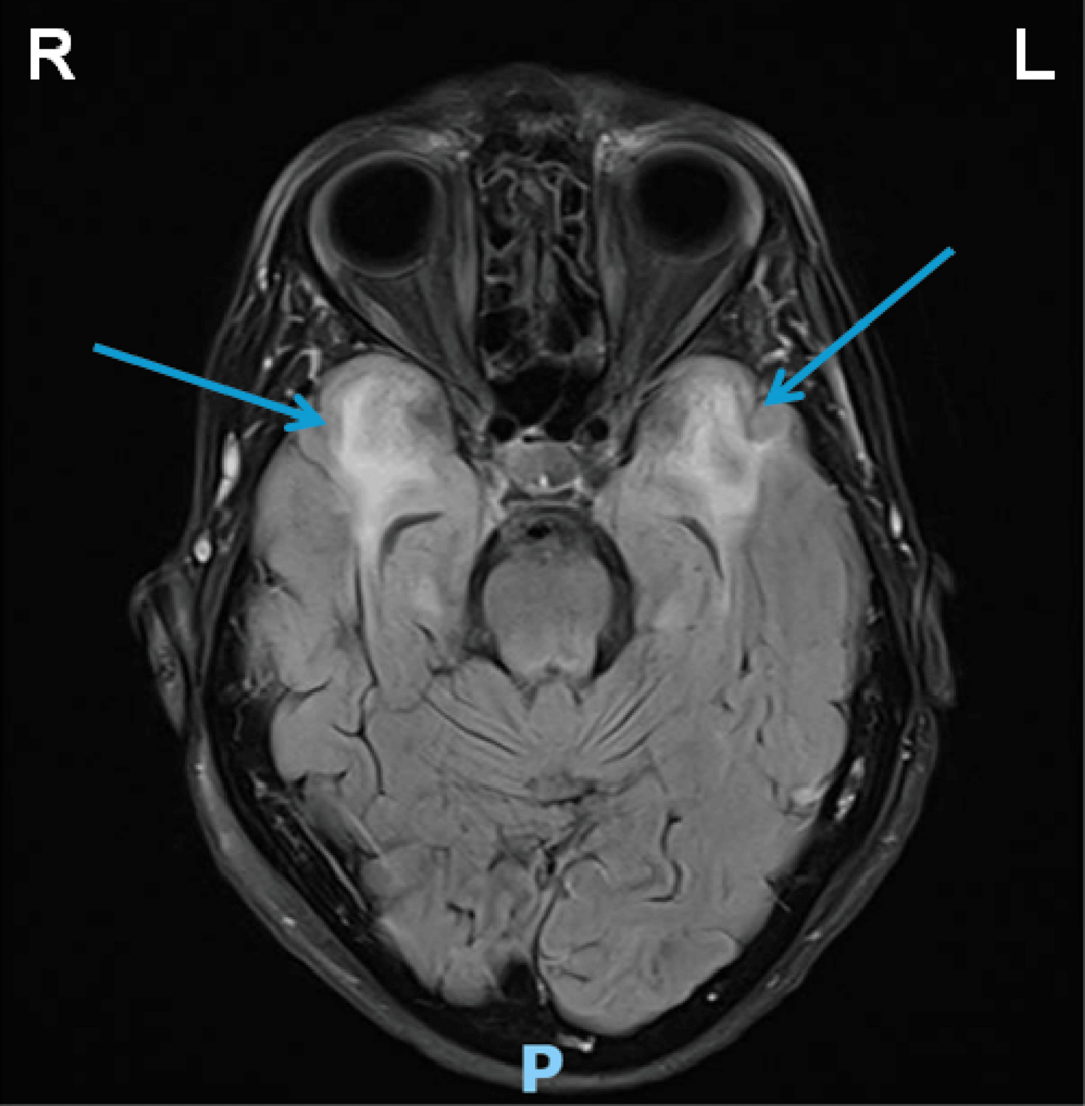
Axial MRI brain demonstrating subcortical arteriosclerotic encephalopathy (blue arrows) MRI: magnetic resonance imaging; CADASIL: cerebral autosomal dominant arteriopathy with subcortical infarcts and leukoencephalopathy Extensive high signal intensity changes in the brain parenchyma, mainly white matter. Involvement of the temporal lobes suggests CADASIL as a possible differential diagnosis

A brain magnetic resonance imaging (MRI) was arranged to confirm the diagnosis of acute stroke. It showed a small acute infarct in the white matter of the right frontal lobe (Figures 2-3) as well as signal intensity changes, which were described as non-specific but most likely representing subcortical arteriosclerotic encephalopathy (Figure 4).

The involvement of the temporal lobes suggested CADASIL as a likely differential diagnosis. The MRI brain findings were discussed at a weekly Stroke Neuroradiology Multidisciplinary Team Meeting (MDT). It is recommended that genetic testing for CADASIL is appropriate. NOTCH3 gene analysis was arranged at a regional specialist centre Genetic Testing Unit. The centre identified the patient to be heterozygous for a pathogenic NOTCH3 gene variant. The implication from this result is that any offspring of the patient would have a 50% chance of inheriting the pathogenic variant. The patient and her immediate family were recommended to undergo genetic counselling and appropriate follow-up.

In view of her age, a young stroke blood profile was completed, consisting of lupus screen, anti-cardiolipin antibodies, HbA1C, and lipid profile. These were negative. Her serum cholesterol was 6.7 mmol/L (normal for the testing laboratory, <5.2 mmol/l). She was started on atorvastatin, 20 mg, orally, daily. An ultrasound duplex scan of the carotid arteries showed no significant stenosis. She had routine outpatient follow-up for six to 12 weeks, and further appointments can be arranged depending on her clinical progress.

## Discussion

Cerebral SVD includes a spectrum of clinical, neuroimaging, and neuropathological features. Its pathogenesis remains largely unclear but is thought to result from pathological changes in small perforating cerebral arterioles, capillaries, and venules [1,11,12]. SVD plays a significant role in cerebrovascular disease and is a major contributor to cognitive decline, vascular dementia, age-related depression, and gait deterioration [1,6,10]. Although sporadic SVD is most commonly associated with arteriosclerosis due to aging, hypertension, and other conventional vascular risk factors, and cerebral amyloid angiopathy (CAA) [1,6], monogenic forms such as CADASIL are increasingly recognised in clinical practice [10]. 

In the present case, our patient presented with dysarthria in the context of significant white matter changes on CT brain and a family history of early-onset stroke. MRI neuroimaging demonstrated a small acute infarct in the white matter of the right frontal lobe, along with extensive subcortical white matter hyperintensities, including temporal lobe involvement, a radiological hallmark strongly suggestive of CADASIL rather than sporadic SVD [12]. Genetic analysis confirmed the presence of a heterozygous pathogenic variant in the NOTCH3 gene, establishing the diagnosis. 

CADASIL is caused by mutations in the NOTCH3 gene on chromosome 19, leading to abnormal accumulation of NOTCH3 protein in vascular smooth muscle cells. This results in progressively impaired cerebrovascular autoregulation, hypoperfusion, ischaemia, and white matter damage [12]. More than 200 NOTCH3 mutations have been identified, with cysteine-altering variants being the most pathogenic [9,10]. 

CADASIL is the most common monogenic form of SVD, with an estimated prevalence of approximately five per 100,000 in Northern England [10]. Although CADASIL was once considered rare, recent population-based studies have identified NOTCH3 mutations more frequently than expected, suggesting substantial underdiagnosis in clinical practice [8-10]. This highlights the importance of recognising characteristic radiological features and family history, even in older individuals, to ensure timely genetic testing and counselling. 

The clinical manifestations of CADASIL are highly variable, usually beginning in adulthood but sometimes presenting later in life [12]. This aligns with our patient’s presentation at 69 years of age. Clinically, CADASIL may present with a constellation of migraine, cerebrovascular events, psychiatric symptoms, cognitive decline, and progressive dementia [10], and our patient’s history of migraine with aura and stroke reflects the classic presentation. 

Currently, the management of CADASIL remains supportive, focusing on vascular risk reduction. Antiplatelet therapy is commonly prescribed for secondary stroke prevention; however, evidence of its efficacy in CADASIL is limited due to the non-atherothrombotic nature of the vasculopathy, and the risk of haemorrhagic complications must be carefully considered due to the presence of microbleeds in a considerable percentage of CADASIL patients [12]. The appropriateness of antiplatelet use in the management of strokes secondary to CADASIL is undetermined, and so the safety of their use needs to be clarified [12]. Given the evidence that individuals with vascular risk factors, particularly smoking and hypertension, tend to experience a more severe disease course, managing these risk factors is a crucial aspect of CADASIL management [12]. Statins may be indicated in patients with dyslipidaemia, although their effect on disease progression is uncertain [6]. 

Genetic counselling is essential due to the autosomal dominant inheritance pattern, as each child of an affected individual has a 50% chance of inheriting the mutation [13]. Counselling must therefore address not only the inheritance risk but also the psychosocial burden and family planning considerations [12]. 

Future research directions include clarifying the genetic basis to improve prognostication, developing therapies that target the underlying vascular pathology, and testing early interventions to preserve cognitive function [10,13]. Ongoing progress in pathogenetic insight, along with large collaborative research efforts, offers hope for better outcomes in CADASIL [6]. 

## Conclusions

This case highlights the importance of maintaining a high index of suspicion for CADASIL in patients presenting with strokes when imaging reveals unusual patterns of white matter hyperintensities and significant small vessel disease, particularly in the presence of migraine and a family history of early cerebrovascular disease. Genetic testing for NOTCH3 mutations should be considered in such cases, and a confirmed diagnosis must be accompanied by appropriate genetic counselling. While no curative treatments currently exist, rigorous management of vascular risk factors and lifestyle modification remain the cornerstone of care. Continued research into the genetic and vascular mechanisms of CADASIL is essential to enable more accurate prognostication and the development of disease-modifying therapies. 

## References

[1] Pantoni L (2010). Cerebral small vessel disease: from pathogenesis and clinical characteristics to therapeutic challenges. Lancet Neurol.

[2] van der Flier WM, van Straaten EC, Barkhof F (2005). Small vessel disease and general cognitive function in nondisabled elderly: the LADIS study. Stroke.

[3] Herrmann LL, Le Masurier M, Ebmeier KP (2008). White matter hyperintensities in late life depression: a systematic review. J Neurol Neurosurg Psychiatry.

[4] de Laat KF, Tuladhar AM, van Norden AG, Norris DG, Zwiers MP, de Leeuw FE (2011). Loss of white matter integrity is associated with gait disorders in cerebral small vessel disease. Brain.

[5] Gorelick PB, Scuteri A, Black SE (2011). Vascular contributions to cognitive impairment and dementia: a statement for healthcare professionals from the American Heart Association/American Stroke Association. Stroke.

[6] Markus HS, de Leeuw FE (2023). Cerebral small vessel disease: recent advances and future directions. Int J Stroke.

[7] Kalaria RN, Kittner SJ (2020). Top-NOTCH3 variants in the population at large. Stroke.

[8] Rutten JW, Dauwerse HG, Gravesteijn G (2016). Archetypal NOTCH3 mutations frequent in public exome: implications for CADASIL. Ann Clin Transl Neurol.

[9] Hack RJ, Rutten JW, Person TN (2020). Cysteine-altering NOTCH3 variants are a risk factor for stroke in the elderly population. Stroke.

[10] Marini S, Anderson CD, Rosand J (2020). Genetics of cerebral small vessel disease. Stroke.

[11] Wardlaw JM, Smith C, Dichgans M (2013). Mechanisms of sporadic cerebral small vessel disease: insights from neuroimaging. Lancet Neurol.

[12] Di Donato I, Bianchi S, De Stefano N (2017). Cerebral autosomal dominant arteriopathy with subcortical infarcts and leukoencephalopathy (CADASIL) as a model of small vessel disease: update on clinical, diagnostic, and management aspects. BMC Med.

[13] Hack RJ, Rutten J, Lesnik Oberstein SA (2000). CADASIL. In: Adam MP, Feldman J, Mirzaa GM, et al., editors. GeneReviews® [Internet], Seattle (WA): University of Washington, 2000. [updated 2019 Mar 14; cited 2025 Oct 9].

